# Impact of sensitisation workshop on empathy levels among postgraduate students: A pre-post interventional study

**DOI:** 10.6026/973206300222659

**Published:** 2026-04-30

**Authors:** Shabana Sultan, Poorva Badkur, Pawan Pandey

**Affiliations:** 1 Department of Obstetrics and Gynaecology, Gandhi Medical College, Bhopal, Madhya Pradesh, India; 2 Department of Community Medicine, Sukh Sagar Medical College, Jabalpur, Madhya Pradesh, India

**Keywords:** Empathy, medical education, Jefferson scale of empathy, postgraduate training, patient-centred care, educational intervention

## Abstract

Empathy plays a vital role in patient-centered care, especially in obstetrics and gynaecology, where patients frequently encounter sensitive and emotionally challenging circumstances. This single-center pre-post interventional study involved 75 postgraduate students from the Department of Obstetrics and Gynaecology at Gandhi Medical College, Bhopal. The intervention was a one-day structured program featuring interactive lectures, educational videos, scenario-based group activities and role-plays. Initial empathy scores were notably low (mean 88.2 ± 7.8). Post-workshop, scores rose markedly to 124.3 ± 8.1 (p < 0.0001). At the eight-week mark, scores decreased to 107.8 ± 9.4 but stayed significantly above baseline (p < 0.0001). The percentage of students achieving high-empathy status (scores ≥116) grew from 21.33% pre-workshop to 33.3% at follow-up.

## Background:

Empathy is an essential part of good medical care [1]. Optimal empathy level among physicians supports trust, better communication and improved clinical outcomes [2]. In obstetrics and gynaecology, empathy becomes even more important because patients often face sensitive, stressful and emotional situations [3, 4]. A supportive and understanding approach from healthcare providers can reduce anxiety and improve both the experience and outcomes of care [5]. However, despite its importance, several studies have concluded that empathy often declines during medical training [6, 7]. Heavy workloads, stressful clinical duties and emotional fatigue can reduce a trainee's ability to connect with patients [8]. Medical education has traditionally focused on imparting technical knowledge and skills [9]. Empathy has not always been taught as a core clinical skill [10]. In recent years, there has been growing interest in including structured empathy training in medical curricula [11]. Workshops, role-plays and reflective activities have shown promise in strengthening empathetic skills and improving patient-centred attitudes [12]. Therefore, it is of interest to determine the effect of One-Day Sensitisation Workshop on Empathy Levels among Postgraduate Students of department of Obstetrics and Gynaecology

## Materials and Methods:

A single centre, pre-post intervention study was undertaken as a part of the curriculum innovation project [13]. The study was conducted in the Department of Obstetrics and Gynaecology, Gandhi Medical College, Bhopal, Madhya Pradesh. The total duration of the study was six months divided into three phases of planning (1 month), implementation (4 months) and data analysis & report writing (1 month). The study included a total of 75 first-second-and third-year postgraduate residents in the Department of Obstetrics and Gynaecology. The research protocol was approved by the study institute. The Jefferson Scale of Empathy (Student Version), a validated 20- item Likert scale, was used to assess empathy levels [14]. Socio-demographic information and participant feedback were collected using a semi-structured questionnaire. The data collection process involved.

## Participant recruitment:

Eligible postgraduate residents were identified and approached by the principal investigator.

## Informed consent:

Written informed consent was obtained from participants using a bilingual consent form (Hindi and English).

## Baseline assessment:

Empathy level was assessed using the Jefferson Scale of Empathy (Student Version), a validated and widely used tool for measuring empathy in healthcare trainees. The scale consists of 20 items rated on a 7-point Likert scale, ranging from strong disagreement to strong agreement. Higher total scores indicated higher levels of empathy. The assessment was carried out at three time points. First, baseline empathy levels were measured one-day before the sensitisation workshop. Participants completed the Jefferson Scale individually in a quiet environment. Next day, after the workshop, the same scale was administered again to measure the direct impact of the training. A third assessment was conducted eight weeks after the workshop to evaluate retention of empathy skills. In addition to empathy scores, socio- demographic information was collected using a semi-structured questionnaire to explore whether characteristics such as year of training, gender, marital status, or place of residence influenced changes in empathy.

## Intervention:

The intervention in this study was a structured empathy sensitisation workshop designed to enhance the participants' understanding of patient emotions and to strengthen their communication skills. The workshop was developed after reviewing existing literature on empathy training and identifying key elements that are known to improve empathetic behaviour in clinical settings [15, 16-17]. It was delivered as a single day, 7 hours, well- planned session using multiple interactive teaching-learning methods. The workshop began with a series of 3 interactive lectures that introduced the concept of empathy, its role in patient care and the evidence linking empathy to improved clinical outcomes. Following the lectures, educational videos were shown to illustrate real-life scenarios where empathy influences patient experiences. These videos were selected to highlight non-verbal communication, emotional cues and the consequences of empathetic and non-empathetic interactions. Role- play formed a core component of the intervention. Students were divided into small groups and asked to perform scenario-based role-plays that reflected common situations in obstetrics and gynaecology. Each role-play was followed by group reflection and feedback, allowing participants to discuss challenges and identify areas for improvement. In addition, group activities were conducted using clinical scenarios that required students to work together, recognise patient emotions and propose empathetic responses.

## Follow-Up assessments:

Empathy levels were reassessed next day after the workshop and at eight weeks post-workshop.

A total of 5 such workshops were held on different dates with 15 participants included in each workshop.

## Statistical analysis:

The data were entered into MS Excel and analyzed using State software version 17.0. Empathy scores from the Jefferson Scale of Empathy (Student Version) were treated as continuous variables. Empathy scores at each time point (pre-workshop, immediately post-workshop and eight weeks post-workshop) were summarised using means and standard deviations. Socio-demographic characteristics such as year of training, gender, marital status and place of residence were summarised using frequencies and percentages. The main outcome was the change in mean empathy scores over time. Paired t-tests were used to compare pre-workshop scores with immediate post-workshop scores and eight-week follow-up scores. Two-sided p-values were reported and a p-value of less than 0.05 was taken as statistically significant.

## Results and Discussion:

Empathy is a vital skill for medical practitioners, directly influencing patient satisfaction, treatment adherence and overall clinical outcomes [18, 19]. This study examined the effects of a one-day structured empathy workshop on empathy levels among postgraduate medical students. All 75 postgraduate students from the Department of Obstetrics and Gynaecology participated and completed the study. Most participants were female and the majority were unmarried and residing in the hostel Table 1 (see PDF). Before the workshop, the mean Jefferson Scale of Empathy score was 88.2 ± 7.8. Baseline empathy scores in this group were markedly low, revealing a significant shortfall in empathetic competencies for resident trainees. Such deficits may stem from intense clinical demands, chronic sleep deprivation and ongoing exposure to traumatic scenarios. This study was conducted at a high-volume tertiary care referral hospital managing roughly 1,200 deliveries, 600 C-sections and 7,000 outpatient visits (encompassing antenatal care, gynaecology, family planning and oncology) monthly, these rigorous conditions emotionally and mentally deplete students, fostering a progressive decline in empathy. In a study among Indian medical students, the mean baseline empathy score was reported as 96.01 ± 14.56, which is slightly higher than our findings [20]. Another nationwide study conducted in USA have also reported significantly higher baseline empathy scores (mean of 116.54 ± 10.85) [21]. These disparities in baseline empathy scores may stem from cultural differences in empathy expression, variations in medical curricula that emphasize humanistic education, or lower burnout rates in some Western programs. Table 2 (see PDF) shows the change in empathy score during the study. The empathy sensitisation workshop in this study resulted in a substantial immediate improvement in empathy levels among postgraduate students (JSE scores rising from 88.2 at baseline to 124.3 post- intervention, representing an approximate 41% increase). A study involving Greek medical students in a 20-hour intensive experiential empathy training reported a more modest increase of 11.25 points (from 106.55 ± 11.118 to 117.85 ± 7.451, p < 0.001) [22]. Likewise, a Nepalese investigation of first-year medical students exposed to a 16-hour Medical Humanities module documented a 10.77-point rise (from 105.52 ± 10.45 to 116.29 ± 9.02, p < 0.001) [23]. In an Ethiopian cluster-randomized trial among healthcare providers (predominantly nurses), a three-day empathy training yielded an 11% increase (approximately 11.52 points, from 101.13 ± 17.67 to 112.65 ± 18.99) one week post-intervention [24]. Before the workshop, 44.0% of participants fell into the low empathy category (scores ≤100). This proportion dropped sharply to 13.3% immediately after the workshop and remained lower than baseline at 17.3% at the eight-week follow-up. Participants in the high empathy category (scores ≥116) increased from 21.33% pre-workshop to 46.7% immediately after the workshop, but this proportion decreased to 33.3% at the eight-week follow-up Table 3 (see PDF). At the eight-week follow-up, scores declined to 107.8 ± 9.4 but remained significantly elevated above baseline (p < 0.0001), indicating a 22% sustained improvement. These findings demonstrate that empathy can be effectively bolstered through targeted interventions. The partial decline at eight weeks echoes trends in other research, highlighting the challenge of sustaining empathy without repeated exposure. The Greek study noted a reduction to a 6.514-point net gain at six months (112.66 ± 8.951, p < 0.005) [22]. Similarly, another study observed progressive decay, with gains dropping to 8% at one month and 5% at three months [24]. A U.S. study on second-year medical students using video-based interventions found a small initial increase of 2.2 points (from 113.0 ± 11.4 to 115.2 ± 12.3, p ≤ 0.01), which dissipated at 10 weeks without reinforcement. However, the authors also reported that higher empathy levels were preserved (115.7 ± 9.4) in a subgroup of students who received an additional lecture [19]. The observed decline in empathy scores after eight-week follow-up, suggests that the initial gains from the sensitisation intervention may erode without sustained support. Potential reasons for this attenuation include the resumption of high clinical workloads, long hours and time constraints, which limit opportunities for empathetic patient interactions and contribute to emotional exhaustion and burnout [25]. Additionally, unsupportive learning environments, such as overcrowded clinics, hierarchical structures and negative role models, can foster detachment and prioritize technical skills over relational ones. These findings align with broader literature indicating that single-session interventions, though effective short-term, often fail to counteract ingoing environmental pressures. To mitigate such decay, future programs should incorporate follow-up sessions, mentorship and curriculum integration to sustain empathy gains in high-stress fields like obstetrics and gynaecology. Empathy scores increased across all participant subgroups when comparing pre-workshop values with the scores recorded eight weeks after the intervention Table 4 (see PDF). Among the three postgraduate training years, baseline scores were similar; however, the greatest improvement was observed in third-year residents. Gender-based comparisons revealed that both males and females experienced improvements, though females consistently scored higher at both time points.

## Conclusion:

This study reveals low initial empathy levels among medical residents, underscoring the necessity for targeted training programs. A structured empathy sensitization workshop significantly enhanced empathy scores, affirming that empathy is a learnable skill bolstered by focused educational interventions. Key findings include demographic variations-such as differences by training stage, gender and living arrangements-that affect training responsiveness. Ultimately, the research emphasizes the importance of continuous reinforcement to sustain long-term empathy improvements.

## Advancement to knowledge:

This study demonstrates that a single, structured, one-day sensitisation workshop can produce a marked and statistically significant rise in Jefferson Scale of Empathy scores, with partial retention at eight weeks. The findings quantify both immediate gain (41% increases) and short-term decay, highlighting the limits of one-time interventions in high-burden clinical settings. The study supports the view that empathy is measurable, teachable and modifiable within postgraduate curricula, while emphasising the need for longitudinal reinforcement strategies rather than isolated sessions.

## Funding:

There was no external funding for this study. All expenses related to the intervention were borne by the study institute and the study participants were not paid any compensation to participate.
